# Taking a Call Is Facilitated by the Multisensory Processing of Smartphone Vibrations, Sounds, and Flashes

**DOI:** 10.1371/journal.pone.0103238

**Published:** 2014-08-12

**Authors:** Ulrich Pomper, Jana Brincker, James Harwood, Ivan Prikhodko, Daniel Senkowski

**Affiliations:** Department of Psychiatry and Psychotherapy, St. Hedwig Hospital, Charité-Universitätsmedizin Berlin, Berlin, Germany; University G. d'Annunzio, Italy

## Abstract

Many electronic devices that we use in our daily lives provide inputs that need to be processed and integrated by our senses. For instance, ringing, vibrating, and flashing indicate incoming calls and messages in smartphones. Whether the presentation of multiple smartphone stimuli simultaneously provides an advantage over the processing of the same stimuli presented in isolation has not yet been investigated. In this behavioral study we examined multisensory processing between visual (V), tactile (T), and auditory (A) stimuli produced by a smartphone. Unisensory V, T, and A stimuli as well as VA, AT, VT, and trisensory VAT stimuli were presented in random order. Participants responded to any stimulus appearance by touching the smartphone screen using the stimulated hand (Experiment 1), or the non-stimulated hand (Experiment 2). We examined violations of the race model to test whether shorter response times to multisensory stimuli exceed probability summations of unisensory stimuli. Significant violations of the race model, indicative of multisensory processing, were found for VA stimuli in both experiments and for VT stimuli in Experiment 1. Across participants, the strength of this effect was not associated with prior learning experience and daily use of smartphones. This indicates that this integration effect, similar to what has been previously reported for the integration of semantically meaningless stimuli, could involve bottom-up driven multisensory processes. Our study demonstrates for the first time that multisensory processing of smartphone stimuli facilitates taking a call. Thus, research on multisensory integration should be taken into consideration when designing electronic devices such as smartphones.

## Introduction

In our environment, we are often confronted with a large number of stimuli that need to be processed and integrated by our senses. Stimuli from different sensory modalities that occur simultaneously and from the same spatial location often provide a processing advantage compared to stimuli that are presented alone, i.e., in a unisensory fashion. Studies using the redundant target effect (RTE) paradigm [1], in which participants are instructed to respond to any stimulus in a continuous stream of unisensory and multisensory inputs, have frequently shown shorter response times (RTs) for multisensory compared to the constituent unisensory stimuli [2], [3].

The RTE for multisensory stimuli can exceed predictions on the basis of probability summations of unisensory stimuli, which has been used as a behavioral marker for integrative multisensory processing [4]–[6]. The vast majority of studies showing integrative multisensory processing in RTs have used semantically meaningless sensory stimuli with well-defined onset characteristics, such as LED flashes [7], sinusoidal tones [8], or short innocuous tactile input [9]. This raises the question whether multisensory RT facilitation effects are also found for basic but meaningful sensory stimuli that derive from real-life objects.

Functional neuroimaging [10], [11] and electrophysiological studies [12], [13] using semantically meaningful stimuli have consistently shown multisensory interactions. This suggests that naturalistic stimuli are integrated across modalities at the neural level. However, RT facilitation effects for stimuli to which we are often confronted in our everyday life have rarely been shown [14], [15]. Stevenson et al. [16] reported RT facilitation effects for the recognition of audiovisual speech compared to the recognition of speech when the constituent unisensory stimuli were presented alone. However, the stimuli in this study were presented on a monitor and through loudspeakers and not from a real person. In another recent study, Sella et al. [15] showed that the processing of semantically congruent visual, auditory, and tactile stimuli in a virtual reality setup can partly improve behavioral performance. Whether multisensory RT facilitation effects would also be found when stimuli were presented directly from everyday-life objects remains unclear. In the present study we presented visual, tactile and auditory smartphone stimuli in a unisensory, bisensory, and trisensory fashion to examine whether multisensory interactions between them facilitate taking a call. We also explored whether prior experience and daily use of smartphones or mobile phones would predict multisensory RT facilitation effects across participants. The absence of such a finding would indicate that these stimuli, similar to what has been previously found for the multisensory processing of semantically meaningless stimuli, are integrated widely automatically, i.e., without requiring prior learning experience.

## Materials and Methods

### Participants

Twenty-five subjects participated in Experiment 1 (mean age: 27.5 years±3.7 years, 15 females). Twenty-three of them also participated in Experiment 2 (mean age: 27.43±3.6 years, 15 females; due to technical difficulties, two participants were unable to perform Experiment 2). All subjects were right-handed and reported normal or corrected-to-normal vision and normal hearing. They provided written informed consent and were reimbursed for participating. The study was approved by the local Ethics Committee of the Charité – Universitätsmedizin Berlin, and conducted in accordance with the Declaration of Helsinki.

### Procedure and stimuli

Both experiments were conducted in a dimly lit soundproof chamber. Participants sat at a table with a cushioned surface on which they rested their right arm. They held the smartphone in their right hand with a relaxed, yet consistent, posture. The smartphone was placed in the center of the visual field at a distance of 52 cm. A Samsung Galaxy S2 smartphone was used to convey unisensory visual (V), auditory (A) and vibrotactile (T) stimuli, as well as bisensory (VA, AT, VT) and trisensory (VAT) stimuli. The experiment was controlled via Matlab (Mathworks) and run on a remote computer. The phone itself was controlled via self-written Android code. Information between the remote computer and the phone were exchanged via a wireless router. The visual stimulus consisted of the smartphone screen lighting up, while the tactile stimulus consisted of the inbuilt vibration function of the smartphone. The auditory stimulus comprised of a traditional ringing telephone tone (http://freesound.org/people/cs272/sounds/77723/). To mask the noise of the vibration motor during tactile simulation, white noise was presented continuously using a small loudspeaker that was placed on the table. The noise level was set so that it completely masked the motor noise of the smartphone.

**Figure 1:**
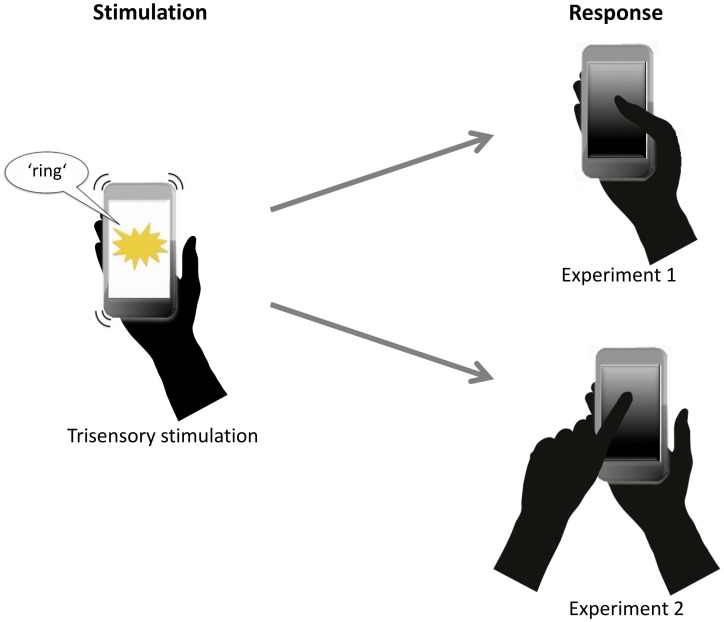
Illustration of Experiments 1 and 2. Participants were presented with different unisensory and multisensory smartphone stimuli. They were instructed to touch the phone's display using either the stimulated hand (Experiment 1, upper right row) or the non-stimulated hand (Experiment 2, lower right row) in response to the appearance of any stimulus.

Prior to the experiment, participants were asked to match the subjectively experienced intensities of visual and auditory stimuli with that of the tactile stimulus. In accordance with the principle of *inverse effectiveness* [17], [18], tactile stimulus intensity was kept at a low level. During the matching of the stimulus intensities, participants held the smartphone in the same position as during the experiments. Pairs of stimuli were presented sequentially, starting with the tactile stimulus followed by either the visual or auditory stimulus. Participants had to verbally report whether the second stimulus was stronger, weaker, or approximately the same intensity as the vibration. Stimulus intensities of visual and auditory inputs were increased and decreased in a step-wise manner until a level was found at which they were rated as being similar to the intensity of the tactile stimulus in three consecutive trials. The mean visual stimulus intensity was 0.173 cd/m^2^ and the mean auditory intensity was 57.5 dB. Each sensory stimulus (i.e. visual, auditory and tactile) lasted for 200 ms and each stimulus was followed by a variable inter-stimulus interval (measured from the offset of a trial to the onset of the next) ranging between 1500 to 3500 ms (mean = 2500). In the bisensory and trisensory conditions, the respective sensory inputs were presented simultaneously (Fig. 1).

### Experiment 1

**Figure 2:**
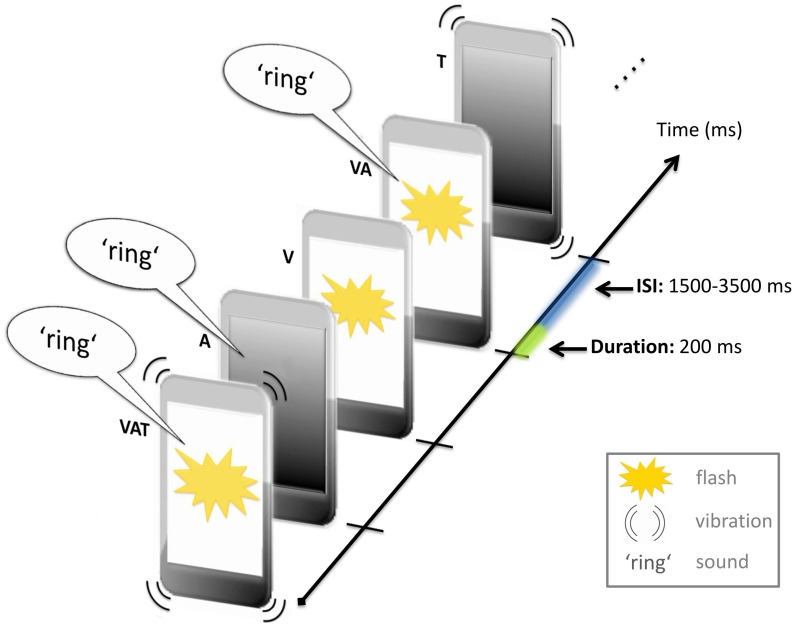
Illustration of the stimulation sequence. A continuous stream of unisensory visual (V), auditory (A), and tactile (T), bisensory VA, VT, VA, and trisensory VAT stimuli was presented in random order.

The experiment consisted of a randomized stream of seven conditions (unisensory V, A, T, and bisensory VA, AT, VT, as well as trisensory VAT). A total of seven blocks with 70 trials each was presented (10 trials per stimulus condition). Participants could initiate each block by touching the screen. They were asked to respond as quickly as possible to any type of stimulation by pressing the screen once with their right thumb (Fig. 2, right upper panel). Participants were also asked to keep their thumb in a relaxed but stable position, at a consistent distance from the screen.

### Experiment 2

A second experiment was conducted to examine whether the fact that the responses were made with the same hand that received tactile inputs, as it was the case in Experiment 1, would influence the results. In addition, we explored whether individual habits in using smartphones in real-life, as obtained by items B8–B10 and C4–C5 of the questionnaire (see Smartphone Questionnaire S1), may differentially influence the multisensory RT effects in the two experiments. The setup, task and number of trials in Experiment 2 were identical to Experiment 1. However, participants were asked to respond with the index finger of their left hand (Fig. 2, right lower panel).

### Questionnaire

Prior to the main experiments participants filled out a questionnaire that consisted of 45 items (see Smartphone Questionnaire S1). This questionnaire served to gather information about the participant's phone use and prior experiences with smartphones (e.g., whether they owned a smartphone or not; frequency of use, time spent using the smartphone for various activities, e.g. telephoning, SMS, browsing the internet, etc.). The questionnaire also included Likert-scaled items probing into the participant's attitudes towards smartphones (e.g., ‘my smartphone is important for my social life’, or ‘most of my smartphone use is work-related’). The questionnaire served to explore whether prior learning experience and current use of smartphones might be related to multisensory integration effects obtained in the two experiments.

### Data analysis

To remove outliers only trials in which participants responded between 100 and 1000 ms were entered into the analysis. After removal of these trials, the remaining trials in which the RT exceeded ±2.5 times the standard deviation of the participant's mean RT for each condition were removed. In the first step of the analysis, it was tested whether there were any differences in RTs between conditions. A repeated measures analysis of variance (ANOVA) was computed using the factor Condition (V, A, T, VA, VT, AT, VAT). Next, follow-up Bonferroni-corrected post-hoc t-tests were calculated between each multisensory condition and the fastest of the constituting unisensory conditions. In the second step of the analysis the ‘Race Model’ [1] was calculated to examine whether RT facilitation by multisensory stimulation exceeds the one predicted by probability summation of unisensory stimuli. The race model determines an upper limit of the cumulative probability (CP) of RTs for multisensory stimuli. At any latency, the race model holds when this CP value is less than or equal to the sum of the CP from each of the constituent unisensory stimuli (CP(t)AV≤((CP(t)A+CP(t)V)). To test the race model, RT data from all conditions were binned into 20 equally sized percentiles, starting from the first percentile (1–5%, 5–10%, …, 90–95%, 95–100%). The percentile boundaries were established by pooling all data relevant to a multisensory condition, and dividing it into percentiles. These boundary values were then applied to each condition separately. For example, for condition VA, the RTs for V, A, and VA were pooled together, divided into percentiles, and the resulting boundary values were then applied to V, A, and VA, respectively. However, to obtain the CP for the trisensory condition (VAT), one cannot sum up the CPs of all three unisensory RTs, as the sum would exceed 1. In line with Diederich and Colonius (2004) we calculated the three trisensory CP estimates by adding to each bisensory CP the remaining unisensory CP (i.e. CP(t)VA + CP(t)T; CP(t)VT + CP(t)A; CP(t)AT + CP(t)V). The empirical trisensory CP was then compared to each of the three combinations. Finally, ‘Miller's inequality’ was calculated as the difference between the CP of the actual multisensory RTs (i.e. empirical data) and the sum of the CPs of unisensory RTs (i.e. race model). For statistical comparison, paired t-tests were conducted between the empirical data and the race model for each percentile. To account for type 1 error accumulation, only percentiles ranging up to 25% were considered (i.e. the first five bins). In line with Kiesel et al. [4], we defined that the majority of the comparisons (i.e. at least three bins) must yield CIs above zero to meet the criterion for race model violation. Any violation of the race model would indicate that the RT facilitation was at least partially due to interactions between the auditory and visual inputs. For all statistical tests, 95% confidence intervals (CIs) and standardized effect sizes (Cohen's d) are reported.

## Results

### Redundant target effect

**Figure 3:**
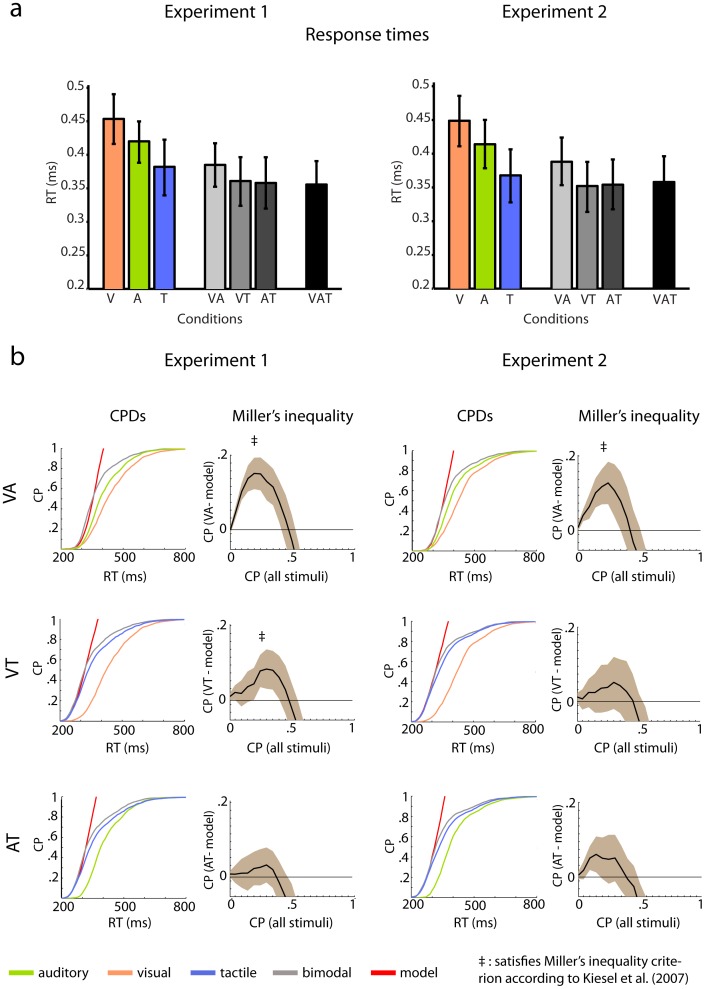
Response times (RTs) for all stimuli, and cumulative probability distributions and Miller's inequality for unisensory and bisensory stimuli. a) RTs to unisensory, bisensory, and trisensory stimuli in Experiment 1 (left panel) and Experiment 2 (right panel). In both experiments RTs were shortest for tactile stimuli and shorter for auditory than for visual stimuli. Moreover, RTs to bisensory stimuli were shorter than the RTs to the respective unisensory constituents. However, RTs to trisensory stimuli did not differ from the responses of the fastest bisensory stimulus combination (i.e. AT stimuli). b) Cumulative probability distributions and Miller's inequality for unisensory stimuli and bisensory stimulus combinations in Experiment 1 (left panel) and Experiment 2 (right panel). Following the criterion by Kiesel et al. (2007), violations of Miller's inequality were found for VA stimuli (upper column) in both experiments and for VT stimuli in Experiment 1 (middle column). No significant violations were observed for AT stimuli.

Fig. 3a illustrates RTs to uni- and multisensory stimuli. The repeated measures ANOVA yielded a significant effect of Condition (F(6,132) = 106.7, p = 0.000), indicating that RTs differed between the seven stimulation conditions. Follow-up -tests were calculated between each multisensory condition and the fastest of its constituting unisensory conditions. Table 1 provides an overview on the outcome of these tests. In both experiments, significant RTE effects were found for all bisensory compared to the unisensory conditions. In Experiment 1 the mean RT was 20.6 ms shorter in the T compared to the VT condition (13.1, 28.2, 95% CI). Moreover, the RT was 23 ms longer for the T condition than for the bisensory AT condition (12.2, 33.7, 95% CI). Finally, the RT was 33 ms longer for the A compared to the VA condition (24.8, 41.3, 95% CI). In Experiment 2 the RT was 15.5 ms longer for the T than for the AT condition (8.1, 23, 95% CI). In addition, the RT was 13.6 ms longer for the T than for the AT condition (4, 23.4, 95% CI). Lastly, the RT was 25.5 ms longer for the A condition compared to the VA condition (18.3, 32.6, 95% CI). In both experiments no significant effects were found for the trisensory VAT condition.

**Table 1:** Redundant target effects: statistical outcome of post-hoc t-tests between RTs to bimodal and unimodal stimulation as well as trimodal and bimodal stimulation.

	condition	mean RT difference (ms)	Cohen's d	t	p (Bonf. corr)	lower CI (95%)	upper CI (95%)
*Experiment 1*	**VT vs T**	20.6	0.23	5.6	0.000	13.1	28.2
	**AT vs T**	23	0.25	4.4	0.001	12.2	33.7
	**VA vs A**	33.0	0.45	8.3	0.000	24.8	41.3
	**VAT vs AT**	2.4	0.02	0.9	1	−3.1	7.9
*Experiment 2*	**VT vs T**	15.5	0.18	4.3	0.001	8.1	23
	**AT vs T**	13.6	0.15	2.9	0.032	4	23.3
	**VA vs A**	25.5	0.29	7.4	0.000	18.3	32.6
	**VAT vs VT**	−5.3	0.06	−2.8	0.041	−10.2	−1.5

We further explored whether the difference in response hand between experiments influences RTs to unisensory tacile stimuli. We found that participants responded faster to these stimuli in Experiment 2 compared to Experiment 1 (t = 2.16, p<0.041).

### Race model and empirical cumulative probabilities (CPs)

**Figure 4:**
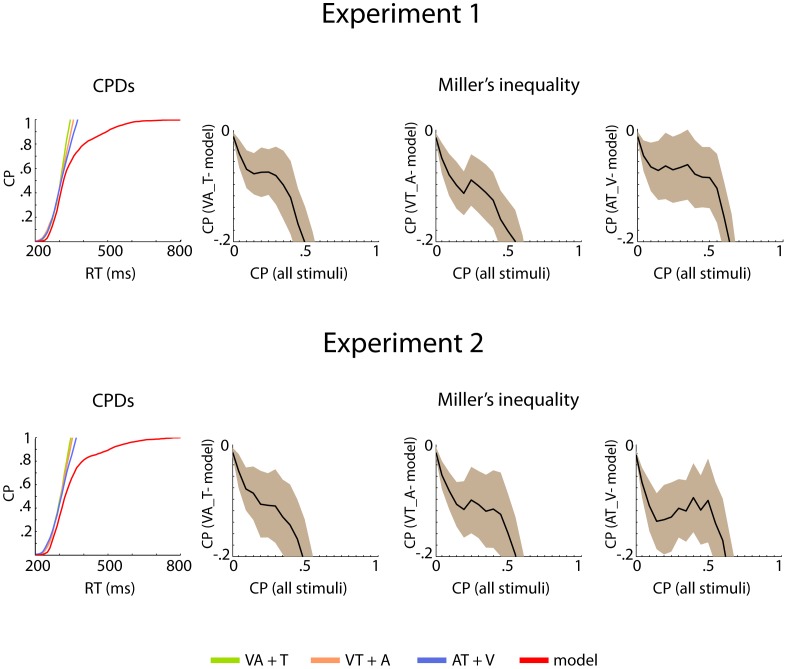
Cumulative probability distributions and Miller's inequality for bisensory and trisensory stimuli. The comparison of bisensory vs. trisensory stimuli did not reveal significant race model violations.

Fig. 3b shows the mean CP distributions of unisensory, bisensory, and model data, as well as the resulting Miller's inequality for all bisensory conditions. Table 2 summarizes the significant results of the t-tests comparing the empirical CP with those of the model for the first five percentiles of all conditions. For Experiment 1, the tests of the race model inequality revealed multisensory RT facilitation effects for the VA and the VT conditions. In condition VA, t-tests between model and empirical CPs revealed significant differences for the first five percentile bins, which were the focus of the presented analysis (Table 2). In condition VT, t-tests between model and empirical CPs revealed significant differences for percentile bins one, four and five (p = 0.034, p = 0.043, p = 0.001, respectively). For Experiment 2, the tests of the race model inequality revealed a multisensory RT facilitation effect in particular for the VA condition. Significant differences were found for the first five percentile bins. In condition AT t-tests between model and empirical CPs revealed significant differences for percentiles two and three but the criterion defined by Kiesel et al. [4], whereby the majority of t-tests need to be significant, was not fulfilled. No significant effects were observed in the VT condition. Finally, in both experiments no significant RT facilitation effects were found in the trisensory condition (Figure 4).

**Table 2:** Miller's inequality: statistical outcome of significant t-tests between cumulative probabilities from empirical data for bimodal stimulation and the corresponding race model.

	condition	percentile	Miller's inequality	Cohen's d	t	p	lower CI (95%)	upper CI (95%)
*Experiment 1*	**VA**	1	4.3	2.36	−5.9	0.000	5.7	2.7
		2	9.7	2.90	−9.3	0.000	11.9	7.5
		3	13.0	3.23	−8.1	0.000	16.4	9.7
		4	14.3	3	−7.4	0.000	18.3	10.3
		5	14.2	2.8	−7.0	0.000	18.4	10.0
	**VT**	1	2.0	0.9	−2.2	0.034	3.9	0.2
		4	4.2	0.85	−2.2	0.043	8.3	0.1
		5	7.5	1.5	−3.8	0.001	11.6	3.4
*Experiment 2*	**VA**	1	3.8	2.23	−5.4	0.000	5.3	2.4
		2	6.1	1.66	−4.0	0.001	9.2	3
		3	9.6	2.19	−5.3	0.000	13.3	5.9
		4	11.2	2.13	−5.2	0.000	15.6	6.7
		5	12.1	1.92	−4.7	0.000	17.5	7.1
	**AT**	2	4.8	1.37	−3.3	0.003	7.9	1.8
		3	5.8	1.09	−2.6	0.016	10.3	1.2

### Multisensory integration effects and smartphone questionnaire items

To investigate a possible relationship between RTEs and everyday smartphone usage, we calculated Pearson's correlation between the mean value of Miller's inequality for percentiles one to five and each item on the smartphone questionnaire. Welch's t-tests (for unequal sample sizes) between mean Miller's inequality values were calculated for binary variables such as gender, phone type (whether the participant's phone was the same type used in the experiment or not) and computer-gamers vs. non-gamers. In Experiment 1 we found a significant negative correlation between the item ‘monthly phone bill’ and the mean Miller's inequality value for condition VA (r = −0.44, p = 0.0262, uncorrected). In addition, a negative correlation between the item ‘daily music listening’ (in minutes) and mean Miller's inequality value for condition VT was observed (r = −0.49, p = 0.0127, uncorrected). In Experiment 2, we found a negative correlation between the item ‘daily music listening’ (in minutes) and the mean Miller's inequality value for condition VA (r = −0.48, p = 0.0199, uncorrected). However, none of these correlations survives Bonferroni correction. For this reason, we hesitate from interpreting and discussing these correlations in further detail. None of the results from Welch's t-tests that was used to examine the nominal scale items of the questionnaire reached significance. Finally, our exploratory analysis of whether habits in using a smartphone in real-life would differentially affect the multisensory RT facilitation effects in the two experiments did not reveal any significant results.

## Discussion

This study investigated multisensory processing of smartphone stimuli when taking a call. A main finding was a robust RT facilitation effect when a ring tone was presented together with a flashing screen. Moreover, multisensory interactions were found when visual and tactile stimuli were presented simultaneously, but only if participants responded with the stimulated hand. The presentation of stimuli in three modalities (i.e. visual, tactile, auditory) at a time did not lead to shorter RTs compared to when stimuli were presented in a bisensory fashion.

The observation of RT facilitation effects for bisensory VA stimuli is in agreement with previous studies using basic sensory stimuli [6]–[8], as well as with studies including more complex stimuli, such as looming cues [2]. In the present experiments, RTs to bisensory audiovisual stimuli were much shorter (on average around 30 ms) than RTs to unisensory auditory and unisensory visual stimuli. The test of Miller's inequality revealed that this response benefit exceeds the benefit that one would predict based on probability summation of unisensory stimuli. This finding demonstrates that it is beneficial to add a flash to the sound, for instance when the smartphone is placed on the desk in front of oneself.

The second main finding of our study was a RT facilitation effect for bisensory VT stimuli. Interestingly, this effect was observed only when participants responded with the hand that was stimulated (i.e. in Experiment 1) but not when they responded with the other hand (i.e. in Experiment 2). A previous study has investigated multisensory RT facilitation in basic VT stimuli [19]. Forster et al. [19] presented unisensory V, unisensory T, and bisensory VT stimuli in a variety of different experimental settings including spatial alignment and non-alignment, stimulation with the stimulated hand being at different distances from the body, and a condition in which the visual stimuli are seen in a mirror. A main observation in this study was that multisensory RT facilitation effects occured in all bisensory stimulation conditions. In the present study, RT facilitation effects for VT stimuli occurred only when the behavioral response was made with the stimulated hand. The lack of integration effects for VT stimuli in the second experiment may be related to the observation of faster RTs when the responses were made with the non-stimulated hand (i.e. Experiment 2) compared to the stimulated hand (i.e. Experiment 1). It may be that the multisensory RTs facilitation effects for bisensory VT stimuli in the first experiment are found due to the specificity of the stimulation device. The vibration of the smartphone stimulated almost the entire hand and was thus a highly salient stimulus. In combination with the response that was required by the same hand, this may have enhanced the sensitivity to uncover RT facilitation for bisensory VT stimuli. Further research is required to address the issue of how stimulus intensities may influence the multisensory processing of smartphone stimuli. Taken together, we found multisensory interactions between visual and tactile stimuli but these effects occurred only when stimulation and response were assigned to the same hand.

In contrast to previous studies, we did not observe RT facilitation effects for bisensory AT [9], [20], [21] and trisensory VAT stimuli [5]. In these other studies, faster responses were found for auditory than for tactile stimuli. Moreover, RTs to bisensory VT and trisensory VAT stimuli were much shorter than the responses to the constituent unisensory stimuli. By contrast, in the present two experiments we found shorter RTs to tactile compared to auditory stimuli. Furthermore, RTs to bisensory AT and trisensory VAT stimuli were only slightly shorter than RTs of unisensory T stimuli. Notably, RTs to bisensory AT stimuli did not significantly differ from RTs to trisensory VAT stimuli. Thus, there was no behavioral benefit when adding a visual input to bisensory AT stimuli. It is possible that a ceiling effect in behavioral performance contributed to the absence of RT facilitation effects for bisensory AT and trisensory VAT stimuli. RTs to tactile stimuli presented alone were already relatively short, especially in comparison to auditory and visual stimuli. In line with the principle of inverse effectiveness [17], [18], we attempted to use low intensity sensory stimuli in the present experiments. However, given that the entire phone vibrated, the tactile stimuli, as well as the intensity matched visual and auditory inputs, were still salient. Future studies that use smartphones, which enable the presentation of very low intensity tactile stimuli may reveal multisensory integration effects between auditory and tactile inputs. Future studies could also examine multisensory interactions between auditory and tactile stimuli in situations in which the phone is not visible. For instance, multisensory interactions may be investigated in a setup where the smartphone is placed in the trouser pocket, while participants perform a visual task. The distracting effect of unisensory tactile and auditory stimuli on visual task performance could be compared with the distraction effect of bisensory AT stimuli. Such a setup would resemble a naturalistic situation in which a driver receives a call while the smartphone is the trouser pocket.

In the present experiments, participants explicitly attended to the sensory stimuli derived by the smartphone. It is known that attention can strengthen multisensory processes [22], [23]. Thus, the present experiments do not allow conclusions about whether the flashing of a smartphone at which we do not directly look would also lead to multisensory RT facilitation effects. In this regard, the overall lack of correlations between smartphone experience and the strength of multisensory integration effects across participants is an interesting observation. The interpretation of statistical null results should be done with great caution. Various other factors, such as lack of power, low signal-noise ration, etc., could contribute to the absence of significant findings. Moreover, albeit stimuli in our study derive from a smartphone, they were presented under strictly controlled experimental conditions. Hence, although we took great care in resembling a naturalistic situation (i.e. taking a call), our experimental setup differs from our everyday life experience with smartphones. This is another factor that could have contributed to the lack of correlations between smartphone experience and the strength of multisensory interactions. In the present case, however, we are tempted to hypothesize that the lack of robust significant correlations could indicate that the observed effects involve, at least in part, bottom-up driven multisensory processes. Previous studies have shown that basic sensory stimuli, which are presented under highly artificial stimulation conditions, can be integrated in a bottom-up fashion [6], [8]. In a similar vein, bottom-up integration mechanisms could have contributed the observed multisensory RT facilitation effects when taking a call. Taken together, this is the first study to investigate multisensory facilitation effects in smartphone stimuli. We found robust multisensory interactions between ring tones and flashes, suggesting that it is beneficial to add a visual flash to the smartphone ringtone, especially when looking at the phone. Multisensory RT facilitation effects were also observed for a vibrating phone that simultaneously flashes but only when the call was answered with the hand in which the phone was placed.

Finally, our analysis, in which we related the strength of multisensory RT facilitation effects with a variety of behavioral parameters, such as prior experience and frequency of smartphone use (see supporting information), did not reveal any robust correlations. This indicates that the observed RT facilitation effects involve, at least to some extent, similar bottom-up mechanisms as previously found for the multisensory integration of basic but meaningless sensory stimuli. Thus, the integration of sensory stimuli that derive from real-world objects likely involves similar mechanisms as the integration of basic sensory stimuli that are often used in experiments studying multisensory processing. Hence, our findings suggest that research on multisensory processing should be considered when designing everyday life electronic devices that provide sensory stimulation.

## Supporting Information

Smartphone Questionnaire S1 **Questionnaire on smartphone use and prior experiences with smartphones.**
(PDF)

Rawdata S1 **Zip file containing the raw data for both experiments.**
(RAR)
