# Supplementary material for: Taking a Call Is Facilitated by the Multisensory Processing of Smartphone Vibrations, Sounds, and Flashes
Source: PLoS One. 2014 Aug 12;9(8):e103238. doi: 10.1371/journal.pone.0103238 (PMC4130528; doi:10.1371/journal.pone.0103238)
Supplement: Smartphone Questionnaire S1 — Questionnaire on smartphone use and prior experiences with smartphones. (PDF) [file pone.0103238.s001.pdf]

Date: __________ Participant: __________

Questionnaire: Multisensory processing of smartphone stimuli

A. Personal information

1. Gender:
    - ☐ female
    - ☐ male

2. Age: _____ years

B. Information about smartphone use

1. Ich own a smartphone / mobile phone
    - ☐ yes
    - ☐ no (please skip to part D)

2. I have owned it for approx. ________ months

    Current model:____

3. If you have switched phones recently, please provide information about the previous model and when you switched:

    Previous model: ____

    Switched ________ months ago

4. My smartphone / mobile phone can connect to the internet
    - ☐ yes
    - ☐ no

5. I use my smartphone / mobile phone on a...
    - ☐ daily
    - ☐ weekly
    - ☐ monthly
    - ☐ less than monthly

...basis

6. If you use your smartphone / mobile phone on a daily basis, how much time do you approx. spend on the the following activities...
    (in minutes)

Telephoning...approx.______mins

SMS..approx.______mins
eMail..approx.______mins
Facebook(and similar)...approx.______mins
WhatsApp(and similar)..approx.______mins
Instagram(and similar)..approx.______mins
Twitter(and similar)..approx.______mins
General surfing the internet...approx.______mins
Games..approx.______mins
Skype...approx.______mins
Music..approx.______mins
Photos..approx.______mins
Managing appointments...approx.______mins
Other (please specify)____ approx.______mins

7. My monthly bill is approx. _________ Euros

8. I usually carry my smartphone / mobile phone...

☐ in my handbag
☐ in my trouser pocket
☐ other (please specify):____

9. While telephoning I usually hold my smartphone / mobile phone...

☐ with my right hand
☐ with my left hand

10. For other activities on my smartphone / mobile phone (e.g. typing, surfing), I usually hold my phone...

☐ with my right hand
☐ with my left hand

C. Information about smartphone settings

A) For calls:

1. Ringtone

☐ Classic ringtone (already installed on the device)
☐ Custom ringtone (e.g. music, sound, etc.)

2. Description of the classic ringtone (for non-personalised calls):
____

3. The volume-strength for my ringtone is set in the

☐ lowest ¼
☐ second ¼
☐ third ¼
☐ highest ¼

...of my phone's volume control bar

4. The vibration function of my smartphone / mobile phone is activated

- ☐ yes
  - ◻ vibrates with ringtone
  - ◻ vibrates without ringtone
- ☐ no

5. The vibration strength is:

- ☐ weak
- ☐ medium
- ☐ strong

B) For SMS and other notifications:

7. Notification tone

- ☐ Classic tone (already installed on the device)
- ☐ Custom tone (e.g. music, sound, etc.)

8. Description of the classic ringtone (for non-personalised calls):

____

9. The volume-strength for my ringtone is set in the

- ☐ lowest ¼
- ☐ second ¼
- ☐ third ¼
- ☐ highest ¼

...of my phone's volume control bar

10. The vibration function of my smartphone / mobile phone is activated

- ☐ yes
  - ◻ vibrates with ringtone
  - ◻ vibrates without ringtone
- ☐ no

11. The vibration strength is:

- ☐ weak
- ☐ medium
- ☐ strong

D. Statements about (my) smartphone(s)

Please circle the number that reflects your opinion most accurately:

1- I fully disagree
2- I partly disagree

3- I don't know
4- I partly agree
5- I fully agree

1. A smartphone is important for maintaining social interaction.

Fully disagree 1 --- 2 --- 3 --- 4 --- 5 Fully agree

2. A smartphone is important work interactions.

Fully disagree 1 --- 2 --- 3 --- 4 --- 5 Fully agree

3. I predominantly use my / a phone in a private capacity.

Fully disagree 1 --- 2 --- 3 --- 4 --- 5 Fully agree

4. I predominantly use my / a phone in a work capacity.

Fully disagree 1 --- 2 --- 3 --- 4 --- 5 Fully agree

5. I often look at my smartphone, even if nothing happens.

Fully disagree 1 --- 2 --- 3 --- 4 --- 5 Fully agree

6. I regularly purchase new smartphone models.

Fully disagree 1 --- 2 --- 3 --- 4 --- 5 Fully agree

7. I use many smartphone apps and regularly download new ones.

Fully disagree 1 --- 2 --- 3 --- 4 --- 5 Fully agree

8. Sometimes I think that my smartphone rings / vibrates, even though it does not.

Fully disagree 1 --- 2 --- 3 --- 4 --- 5 Fully agree

E. Further information

1. During the experiment I employed a specific strategy

☐ yes
☐ no

If yes, please specify:

____

____

2. I play computer / console games.

☐ yes, approx. _______ hours per ______________.
☐ no
